# Appendiceal endosalpingiosis: a rare case with potential implications for gynecological malignancy

**DOI:** 10.1093/jscr/rjaf1024

**Published:** 2025-12-28

**Authors:** Muller Pierre-Louis, Luke Bauerle, Manasa Ponnapalli, Morgan Choma, Syeda F Absar, Thomas Donkar

**Affiliations:** Department of Surgery, St. Luke’s University Health Network, 801 Ostrum Street, Bethlehem, PA 18015, United States; Department of Research and Innovation, St. Luke’s University Health Network, 801 Ostrum Street, Bethlehem, PA 18015, United States; Department of Obstetrics and Gynecology, University of Rochester Medical Center, 125 Lattimore Road Suite 110, Rochester, NY 14620, United States; Department of Obstetrics and Gynecology, Penn State College of Medicine, 700 HMC Crescent Road, Hershey, PA 17033, United States; Department of Pathology and Laboratory Medicine, St. Luke’s University Health Network, 801 Ostrum Street, Bethlehem, PA 18015, United States; Department of Surgery, St. Luke’s University Health Network, 801 Ostrum Street, Bethlehem, PA 18015, United States

**Keywords:** endosalpingiosis, appendicitis, appendectomy, endometriosis, malignancy

## Abstract

Appendiceal endosalpingiosis is an exceedingly rare histopathologic finding with uncertain clinical significance and unknown implications for management. Most often, endosalpingiosis occurs within ovary, fallopian tube, omentum, and uterus. Previous literature has elucidated an association between endosalpingiosis and pelvic malignancies, such as ovarian and uterine cancers. Here, we describe a case of appendiceal endosalpingiosis mimicking acute appendicitis. The clinical significance of this condition as well as the potential for association with gynecologic malignancy requires further investigation to be elucidated.

## Introduction

Endosalpingiosis is a rare condition defined by the presence of benign ectopic fallopian tube-like ciliated epithelial tissue on serosal surfaces of the pelvis and peritoneum [1]. While endosalpingiosis has reportedly been associated with various gynecologic malignancies, very few cases of endosalpingiosis of the appendix have been reported and the current overall understanding of the malignant potential of these lesions is minimal [2, 3]. Herein, we detail a case of presumed acute appendicitis that upon histopathologic review of the appendix was determined to be appendiceal endosalpingiosis.

**Figure 1:**
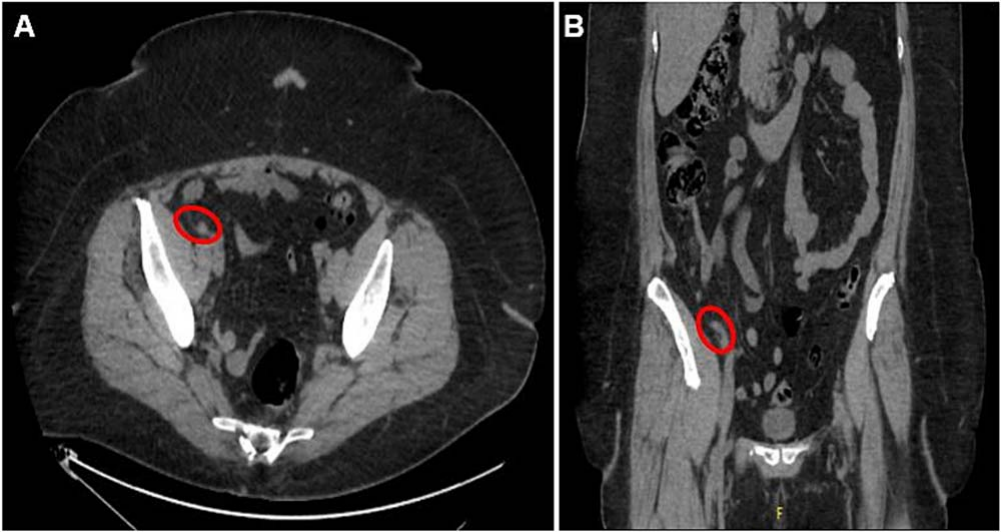
Axial (A) and coronal (B) CT abdomen and pelvis images demonstrating minimally thickened appendiceal tip measuring 7.8 mm with minimal surrounding fat stranding and no adjacent free air or fluid collection, indicating uncomplicated appendicitis.

## Case report

**Figure 2:**
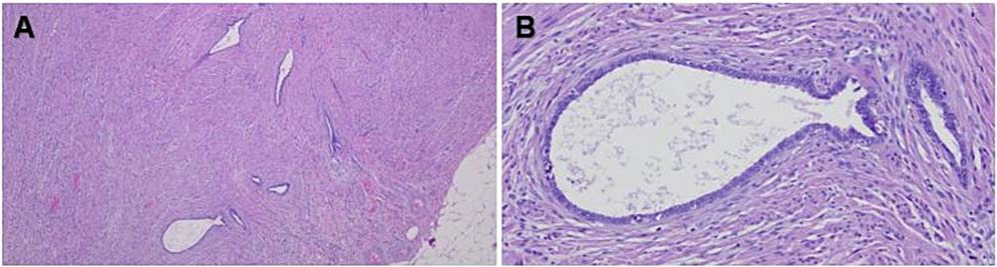
Hematoxylin and eosin (H&E)-stained tissue sections of the appendix showing glandular epithelial structures within the muscularis externa (A) as well as glands and tubules lined by columnar or cuboidal cells with cilia without the presence of endometrial stroma or hemorrhage (B). Original magnification 40× (A) and 200× (B).

**Figure 3:**
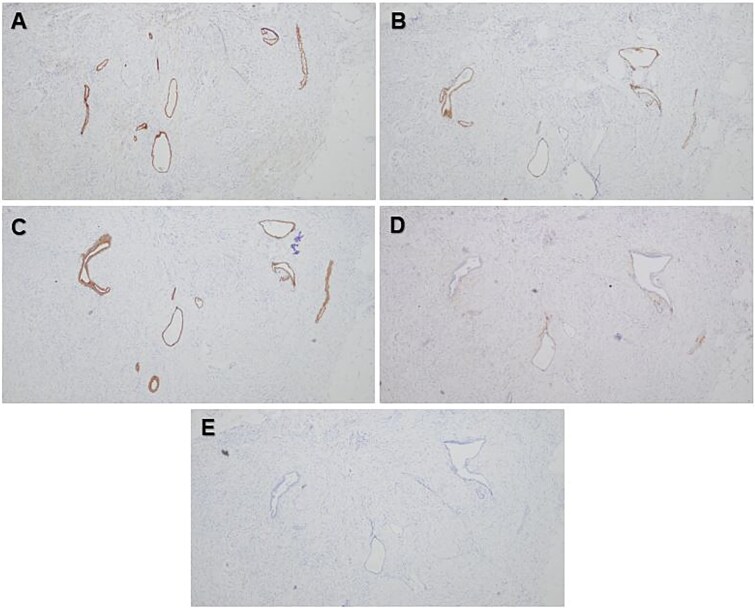
Photomicrographs showing positive epithelial staining with PAX8 (A), ER (B), and CK7 (C) immunohistochemical stains and negative epithelial staining with CD10 (D) and CK20 (E) immunohistochemical stains. Original magnification 40× (A–E).

A 56-year-old female with a history of diverticulitis, obstructive sleep apnea managed with at-home CPAP, hypertension, hyperlipidemia, obesity, asthma, gastroesophageal reflux disease, depression, and a past surgical history of cesarean section and hysterectomy presented to our institution with a four-day history of progressively worsening right lower quadrant abdominal pain associated with decreased appetite and diaphoresis. Patient denied fever, nausea, vomiting, or diarrhea, and laboratory results revealed no leukocytosis, but computed tomography (CT) scans of the abdomen and pelvis revealed minimally thickened appendiceal tip measuring 7.8 mm with surrounding fat stranding suggesting early acute uncomplicated appendicitis (Fig. 1A and B). Given these imaging findings and significant right lower quadrant tenderness to palpation on physical examination, the patient was admitted and subsequently underwent a laparoscopic appendectomy without any intraoperative complications. The patient’s postoperative course was uncomplicated and was eventually discharged on post-operative day 1. Pathological examination of the removed appendix from the operation stained positive for markers PAX8, ER, PR (focal), and CK7 and negatively for markers CD10, CDX2, and CK20, indicating non-cancerous ectopic fallopian tube-type ciliated epithelial tissue consistent with endosalpingiosis of the appendix (Fig. 2A and B, Fig. 3A–E).

## Discussion

Often an incidental finding on histopathologic evaluation, endosalpingiosis has been identified during laparoscopy appearing as peritoneal proliferation, accounting for roughly 7% of peritoneal proliferation findings in premenopausal women [4, 5]. Previous literature has found endosalpingiosis to co-occur with endometriosis; however, it has been demonstrated as a distinct entity with possibly overlapping clinical presentation and gross appearance [6]. In contrast to endometriosis, endosalpingiosis has a greater incidence in postmenopausal women, is less likely to be associated with pelvic pain, and when it does occur in the premenopausal population is significantly associated with incidence of gynecologic malignancy [7]. Recent literature has reported an association between endosalpingiosis and gynecologic malignancy, specifically serous borderline ovarian tumors and ovarian serous tumors of low malignant potential with lymph node involvement [8–11]. These findings however have specifically evaluated peritoneal endosalpingiosis; none to date have associated appendiceal endosalpingiosis with gynecologic malignancy.

Appendiceal endosalpingiosis is exceedingly rare, with only six prior published cases in the current literature [1, 2, 12–15]. It has been described as an appendicitis mimic in that it can cause right lower quadrant pain reportedly without the expected laboratory values and imaging findings associated with acute appendiceal inflammation [1, 2]. This case highlights the overlap in the symptoms of acute appendicitis and endosalpingiosis explored in previous case reports as presentation occurred with progressively worsening right lower quadrant pain and associated malaise and diaphoresis. Differentiation from typical acute appendicitis within this case was the presence of a normal white blood cell count, a similar finding that has been described in at least one previous study reporting a case of appendiceal endosalpingiosis [1]. These findings further suggest that appendiceal endosalpingiosis acts as an acute appendicitis mimic in terms of history and physical exam findings.

Imaging in the presented case demonstrated minimal fat stranding with minimal thickening of the appendiceal tip, suggesting early findings of appendicitis which prompted surgical intervention. This finding in fact contradicts some of the previous literature which proposed a lack of inflammatory processes evident on CT imaging [2]. Additionally, pathological evaluation confirming the complete absence of acute appendicitis suggests that appendiceal endosalpingiosis may induce some inflammatory response visible on imaging, although quite minimal and of unknown significance.

Furthermore, association with gynecologic malignancy cannot be thoroughly evaluated in this case. The patient had undergone total laparoscopic hysterectomy nine years prior to presentation due to indications of post-menopausal abnormal uterine bleeding secondary to an intramural uterine fibroid and chronic pelvic pain. Histopathologic evaluation at that time revealed no evidence of atypia or hyperplasia of the endometrium, normal bilateral fallopian tubes, a benign hemorrhagic luteal cyst of the left ovary, and a small intramural fibroid. Currently, there are no reported rates for endosalpingiosis undergoing appendectomy for suspected appendicitis [2, 3]. There may be a clinically relevant association between the condition and gynecologic malignancy in the setting of acute appendicitis as suggested by previous reports but further research is still required in order to validate this proposed association [2].

Additionally, further questions which arise from the presented case pertain to surveillance and management when appendiceal endosalpingiosis is discovered incidentally. Given this patient’s surgical intervention of total laparoscopic hysterectomy which revealed no evidence of malignancy on histopathological evaluation almost a decade prior to discovery of appendiceal endosalpingiosis, conclusions on potential for malignancy within this patient are limited. Due to the rarity of this finding and sparse data regarding its significance, further investigations are required in order to delineate if an association exists between gynecologic malignancy and appendiceal endosalpingiosis.

In short, we described a rare case of appendiceal endosalpingiosis mimicking acute appendicitis. While there has been an established association between other types of endosalpingiosis and gynecological malignancy, there is limited data to extend this relationship to appendiceal endosalpingiosis. As established in prior reports, it remains unclear as to the clinical relevance and monitoring guidelines for patients who have been diagnosed with appendiceal endosalpingiosis incidentally. The potential association between appendiceal endosalpingiosis and gynecological malignancy must be further explored in order to help guide the future of monitoring and clinical decision-making when this condition is encountered.

## Data Availability

Data sharing is not applicable to this article as no datasets were generated or analyzed during the current study.

## References

[1] Tudor J, Williams TR, Myers DT, et al. Appendiceal endosalpingiosis: clinical presentation and imaging appearance of a rare condition of the appendix. Abdom Radiol 2019;44:3246–51. 10.1007/s00261-018-1813-8 30367212

[2] Nayak A, Karpes J, Stewart K. Appendiceal endosalpingiosis: a case report of a rare finding from appendicectomy. J Surg Case Rep 2022;2022:rjac402. 10.1093/jscr/rjac402 PMC9439693 36072651

[3] Esselen KM, Terry KL, Samuel A, et al. Endosalpingiosis: more than just an incidental finding at the time of gynecologic surgery? Gynecol Oncol 2016;142:255–60. 10.1016/j.ygyno.2016.05.036 27261327

[4] Noor M, Chen A, Gonzalez RS. Clinicopathologic findings in gynecologic proliferations of the appendix. Hum Pathol 2019;92:101–6. 10.1016/j.humpath.2019.08.004 31430494

[5] Hesseling MH, De Wilde RL. Endosalpingiosis in laparoscopy. J Am Assoc Gynecol Laparosc 2000;7:215–9. 10.1016/s1074-3804(00)80043-2 10806265

[6] deHoop TA, Mira J, Thomas MA. Endosalpingiosis and chronic pelvic pain. J Reprod Med 1997;42:613–6. 9350013

[7] Prentice L, Stewart A, Mohiuddin S, et al. What is endosalpingiosis? Fertil Steril 2012;98:942–7. 10.1016/j.fertnstert.2012.06.039 22819185

[8] Lewis GK, Ghaith S, Craver EC, et al. The association of endosalpingiosis with gynecologic malignancy. Gynecol Oncol 2022;167:81–8. 10.1016/j.ygyno.2022.07.025 35909004

[9] Djordjevic B, Clement-Kruzel S, Atkinson NE, et al. Nodal endosalpingiosis in ovarian serous tumors of low malignant potential with lymph node involvement: a case for a precursor lesion. Am J Surg Pathol 2010;34:1442–8. 10.1097/PAS.0b013e3181f17d33 20871218

[10] Clausen I . Peritoneal endosalpingiosis. Zentralbl Gynakol 1991;113:329–32. 2058343

[11] Ryuko K, Miura H, Abu-Musa A, et al. Endosalpingiosis in association with ovarian surface papillary tumor of borderline malignancy. Gynecol Oncol 1992;46:107–10. 10.1016/0090-8258(92)90205-w 1634129

[12] Pollheimer MJ, Leibl S, Pollheimer VS, et al. Cystic endosalpingiosis of the appendix. Virchows Arch 2007;450:239–41. 10.1007/s00428-006-0328-9 17111123

[13] Cajigas A, Axiotis CA. Endosalpingiosis of the vermiform appendix. Int J Gynecol Pathol 1990;9:291–5. 10.1097/00004347-199007000-00009 2373590

[14] Demir MK, Savas Y, Furuncuoglu Y, et al. Imaging findings of the unusual presentations, associations and clinical mimics of acute appendicitis. Eurasian J Med 2017;49:198–203. 10.5152/eurasianjmed.2017.17218 29123444 PMC5665630

[15] Tallón-Aguilar L, Olano-Acosta C, López-Porras M, et al. Endosalpinagiosis apendicular [Endosalpingiosis of the appendix]. Cir Esp 2009;85:383. 10.1016/j.ciresp.2008.09.017 19406386

